# Significance of sampling time on thyroid-stimulating hormone, free thyroxine, and free triiodothyronine levels

**DOI:** 10.5937/jomb0-61454

**Published:** 2026-04-15

**Authors:** Mahmut Mert Daglar, Asuman Orçun

**Affiliations:** 1 Dr. Lutfi Kirdar City Hospital, Department of Biochemistry Laboratory, Istanbul, Turkey

**Keywords:** thyrotropin, thyroid hormones, circadian rhythm, time factors, blood sampling, tireotropin, hormoni štitaste žlezde, cirkadijalni ritam, vremenski faktori, uzorkovanje krvi

## Abstract

**Background:**

The timing of sample collection is crucial for analytes with a circadian rhythm. Although blood samples are usually collected in the morning, they can also be collected in the afternoon. Our study aims to evaluate the impact of sampling time on Thyroid-Stimulating Hormone (TSH), free thyroxine (fT4), and free triiodothyronine (fT3).

**Methods:**

Serum TSH, fT4, and fT3 measurements from 216,278 patients who visited our institute's outpatient clinics over two years (2021-2023) were obtained from laboratory data. After exclusions, 5,953 patient results collected from 8 am to 5 pm were grouped into 1-hour intervals. All measurements were performed using the Cobas e801 (Roche Diagnostics, Penzberg, Germany) with the electrochemiluminescence immunoassay method. Statistical significance was tested with the standard normal deviation test (Z-test), and clinical significance was assessed using bias % values obtained from biologic variation estimates on the website of the European Federation of Clinical Chemistry and Laboratory Medicine (EFLM).

## Abstract (sr)

**Uvod:**

Vreme uzorkovanja ima ključni značaj za analite koji ispoljavaju cirkadijalni ritam. Iako se uzorci krvi najčešće prikupljaju u jutarnjim časovima, uzorkovanje se može obavljati i tokom popodneva. Cilj ove studije je bio da se ispita uticaj vremena uzorkovanja na vrednosti tireostimulišućeg hormona (TSH), slobodnog tiroksina (fT4) i slobodnog trijodtironina (fT3).

**Metode:**

Merenja serumskih koncentracija TSH, fT4 i fT3 kod 216.278 pacijenata koji su u periodu od dve godine (2021-2023) posetili ambulante naše ustanove su preuzeta iz laboratorijske baze podataka. Nakon isključivanja, u analizu je uključeno 5.953 rezultata pacijenata čiji su uzorci prikupljeni u periodu od 8 do 17 časova, grupisanih u jednočasovne intervale. Sva merenja su izvedena na analizatoru Cobas e801 (Roche Diagnostics, Penzberg, Nemačka) primenom elektrohemiluminiscentne imunoanalitičke metode. Statistički značaj je testiran primenom testa standardne normalne devijacije (Z-test), dok je klinički značaj procenjivan na osnovu vrednosti procentualnog odstupanja (bias %) dobijenih iz procena biološke varijacije dostupnih na internet stranici Evropske federacije za kliničku hemiju i laboratorijsku medicinu (EFLM).

**Rezultati:**

Sve jednočasovne grupe TSH između 11 i 17 časova su pokazale statistički i klinički značajne razlike u odnosu na referentnu grupu. Za fT4 i fT3 nisu uočene statistički značajne razlike. Međutim, vrednosti fT4 su pokazale klinički značajnu razliku u jednoj vremenskoj tački kod muškaraca, dok su vrednosti fT3 pokazale klinički značajne razlike u periodu od 15 do 17 časova kod žena, odnosno od 14 do 17 časova kod muškaraca.

**Zaključak:**

Analiza obimnog skupa podataka ukazuje na to da vreme uzorkovanja krvi značajno utiče na rezultate TSH. Ovo može dovesti do pogrešne interpretacije u kliničkoj praksi ukoliko uzorkovanje nije standardizovano.

## Introduction

Serum Thyroid-Stimulating Hormone (TSH) measurement is crucial in diagnosing thyroid hormone disorders but is subject to significant variability, including pre-analytical, analytical, and biological variations (BVs) [1].

TSH levels follow a circadian rhythm, with a peak occurring between 11 pm and 5 am and a nadir between 5 pm and 8 pm. It also has secretory pulses every 2-3 hours with tonic non-pulsatile periods among them [2]. The rhythmicity of thyroid hormones, particularly triiodothyronine (T3), rather than thyroxine (T4), is less clear compared to TSH. T4 levels in circulation are more stable due to its long half-life. In contrast, T3, with its shorter half-life, is expected to partially follow the TSH rhythm, as only a small portion of T3 is directly released from the thyroid [3].

For analytes with circadian rhythms, the timing of sample collection is crucial and must be standardized to ensure accurate clinical interpretation. Physicians need reference data to compare laboratory test results, and they mostly use reference intervals (RIs). RIs are calculated from reference individual values obtained from measurements taken in the morning, usually between 8 and 11 am. In this case, results from samples taken at other times of the day should not be compared with conventional RIs. For such comparisons, RIs derived from samples collected during comparable periods are required. Reference Change Values (RCVs) for analytes are another important tool for clinical decision-making. It enables the physician to assess the clinical significance of the difference between two consecutive measurements. Although it is presented as a powerful tool for monitoring personal serial measurements, the importance of this approach should be reconsidered in light of analyte circadian/ultradian rhythms. Intraindividual variability (CVI), used to calculate RCV, is derived from daily samples measured at the same time of day, typically in the morning. Results from samples taken at different times of day should not be compared with these RCVs [4].

Therefore, understanding the hormonal rhythms and the effects of sample collection time on clinical interpretation are important when comparing patient results with predetermined references such as RIs and RCVs. In diseases such as Subclinical Hypothyroidism (SCH), where the diagnosis is based solely on TSH elevation, the timing of sample collection is important, and consistency in testing conditions is necessary.

According to the Clinical and Laboratory Standards Institute (CLSI), managing clinically important pre-analytical factors is crucial to minimizing their impact on clinical decision-making. Standardizing sampling time will enable both laboratories and physicians to provide accurate clinical assessments [5].

Circadian rhythms of TSH, free thyroxine (fT4), and free triiodothyronine (fT3) have been studied extensively in the medical literature; however, few studies have focused on this rhythm's impact on laboratory and clinical practice. Blood sampling for measurements is commonly performed in laboratories in the morning but is also often done in the afternoon. In this extensive study, involving large sample groups, we aimed to investigate hourly changes in these hormones from 8 am to 5 pm and assess their clinical significance.

## Materials and methods

### Study design

This descriptive data analysis study aims to evaluate the significance of variations in TSH, fT4, and fT3 across different sampling time groups.

### Subjects

**Figure 1:**
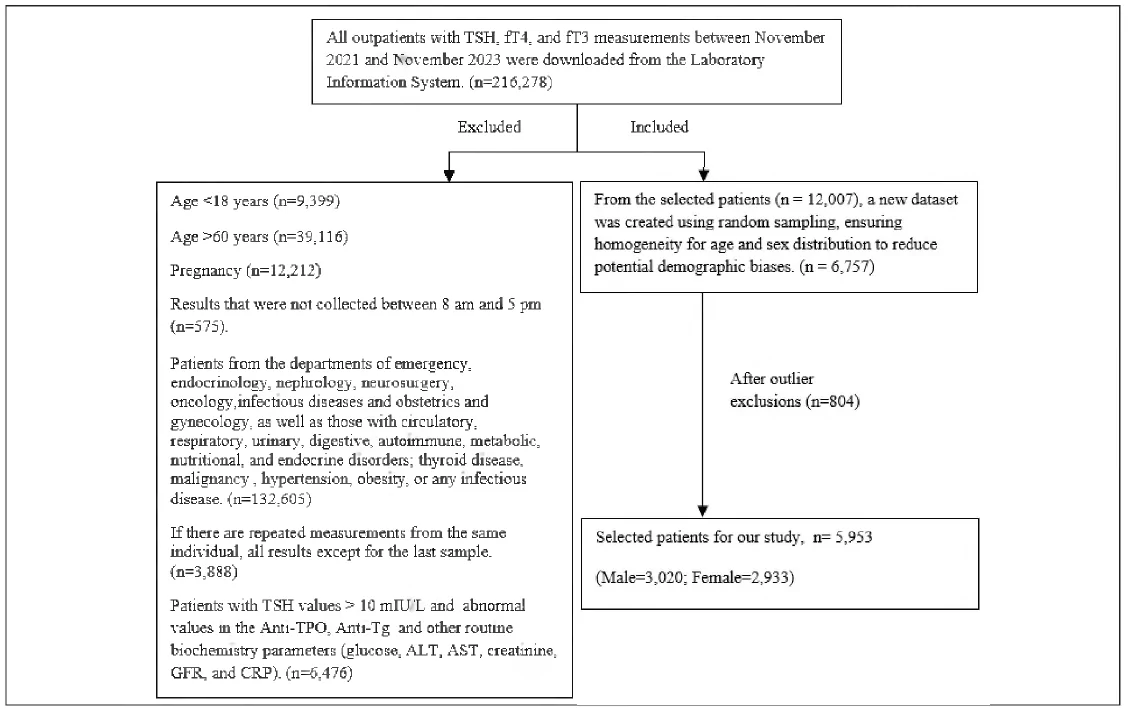
Flow diagram showing the selection of patients.

A total of 216,278 serum TSH, fT4, and fT3 results of patients who visited our institute's outpatient clinics between 2021 and 2023 were retrieved from the Laboratory Information System. Patients under 18 years of age, over 60 years of age, or pregnant patients were excluded from the study. Additionally, patients admitted to the departments of emergency, endocrinology, nephrology, neurosurgery, oncology, infectious diseases, and obstetrics and gynecology, as well as those diagnosed with circulatory, respiratory, urinary, digestive, autoimmune, metabolic, nutritional, or endocrine disorders; thyroid disease, malignancy, hypertension, obesity, or any form of infectious disease were also excluded from the analysis. If there were repeated measurements from the same individual, all results except for the last sample were excluded. Patients with TSH values >10 mIU/L and abnormal values in the Anti-TPO, Anti-Tg, and other routine biochemistry parameters (glucose, ALT, AST, creatinine, GFR, and CRP) were excluded. From the selected patients (n = 12,007), a new dataset (n = 6,757) was created via random sampling, ensuring homogeneity in age and sex distributions to reduce potential demographic biases. A flow diagram of patient selection is provided in Figure 1.

Samples collected during laboratory working hours, from 8 am to 5 pm, were grouped into one-hour interval and nine time points were included in the final dataset as 8-9 am, 9-10 am, 10-11 am, 11 am-12 pm, 12-1 pm, 1-2 pm, 2-3 pm, 3-4 pm, and 4-5 pm. 8-9 am sampling time group was taken as the reference in comparisons (Reference group). Each group was also divided by gender, and comparisons were made for each subgroup.

### Blood sampling

Patient blood samples were collected at all phlebotomy units using BD Vacutainer® SST™ 11 tubes with a serum separator (Becton Dickinson Italia S.p.A., Milan, Italy, ref. no. 366566). The samples were then centrifuged at 3500 x g for 10 minutes, transported to our core laboratory at 0-5°C, and analyzed within 2 hours, as recommended by the manufacturer.

Our study was approved by the Ethics Committee of our institution (Istanbul, Kartal Dr. Lütfi Kirdar City Hospital Ethical Committee; No: 2024/010.99/2/4; 27 March 2024).

### Methods

Analyses for TSH, fT4, and fT3 were performed using the Roche Cobas e801 immunoanalyzer (Roche Diagnostics, Penzberg, Germany) with electrochemiluminescence immunoassay.

Analytical coefficient of variations (CVs) were calculated using laboratory internal quality control data with two levels of control sera, each with two different lots, on four different analyzers, with 2-3 different reagent lots, over six months [6]. The pooled CVs were found as follows: TSH, 3% for level 1 (1.38 mIU/L) and 3.1% for level 2 (8.61 mIU/L); fT4, 3.7% for level 1 (15.44 pmol/L) and 3.5% for level 2 (34.69 pmol/L); fT3, 4.2% for level 1 (5.86 pmol/L) and 3% for level 2 (21.56 pmol/L).

The manufacturer-provided RIs were TSH 0.27-4.20 mIU/L, fT4 12-22 pmol/L, and fT3 3.1-6.8 pmol/L.

### Statistical analysis

Homogeneity of gender, age, and season distribution across time groups was evaluated using the chi-square test, and P<0.05 was considered statistically significant.

The data distribution was evaluated using the Kolmogorov-Smirnov test. Logarithmic transformations were applied to groups with nonGaussian distributions. Outliers were tested for each time group using the Tukey method and subsequently eliminated.

The formula calculated deviation % of hourly groups from the reference group: Dev%= [(Median value of time group - Reference median value) / Reference median value] X100

To evaluate clinical significance, the calculated deviation % values were compared with the recommended analytical performance specifications provided by the European Federation of Clinical Chemistry and Laboratory Medicine (EFLM) for these parameters, as shown in Table 1 [7].

**Table 1:** Minimum, desirable, and optimal bias % values for TSH, fT4, and fT3. Values were taken from the EFLM analytical performance specification database.

Analyte	Minimum<br>Bias %	Desirable<br>Bias %	Optimal<br>Bias %
TSH	15.1	10	5
fT4	3.5	2.3	1.2
fT3	3.6	2.4	1.2

To perform group comparisons, the standard normal deviation test (Z-test) was used, as recommended by CLSI for subgroup partitioning [5]. The calculated Z value was compared with a critical statistic Z value (Z*) according to the formula proposed by Harris and Boyd. Values of Z>Z* were considered statistically significant [8].

Statistical analyses were performed using MedCalc Statistical Software version 22.016 (MedCalc Software Ltd, Ostend, Belgium, 2023).

## Results

After excluding outliers for each time group using the Tukey method, the new dataset included 5,953 patient results from the study: 2,933 female and 3,020 male. The gender, age, and season distributions of patients for each time group are given in Table 2. The distributions of sampling times by gender, age, and season were found to be homogeneous (χ^2^ = 1.78, p=0.98, χ^2^ = 3.10, p = 1, χ^2^=26.84, p=0.31, respectively).

**Table 2:** Demographic characteristics of patients' data according to sampling times. Abbreviations: min = minimum; max=maximum<br>A Homogeneous gender distribution (χ^2 ^= 1.78, p = 0.98)<br>B Homogeneous age distribution (χ^2 ^= 3.10, p = 1)<br>C Homogenous season distribution (χ^2^ = 26.84, p = 0.31)

Sampling Time	n	Male/Female^A^	Median<br>Age<br>(min-max)	Age groups^B^<br>(19-30/31-40/<br>41-50/51-60)	Season groups^C^<br>(Winter/Spring/Summer/<br>Autumn)
8-9 am (Ref group)	908	453/455	40 (19-60)	228/229/226/225	227/228/211/242
9-10 am	1,511	777/734	40 (19-60)	371/387/382/371	414/368/319/410
10-11 am	1,153	593/560	40 (19-60)	291/293/292/277	316/284/215/338
11 am-12 pm	897	455/442	40 (19-60)	221/233/216/227	265/229/173/230
12-1 pm	210	103/107	40 (19-60)	56/55/53/46	59/60/44/47
1-2 pm	285	141/144	40 (19-60)	73/73/73/66	89/77/53/66
2-3 pm	591	292/299	41 (19-60)	149/146/145/151	174/154/107/156
3-4 pm	260	134/126	40 (19-60)	66/65/68/61	75/73/42/70
4-5 pm	138	72/66	40 (19-60)	36/35/34/33	45/32/24/37
**Total**	**5,953**	**3,020/2,933**	**40 (19-60)**	**1,491/1,516/1,489/1,457**	**1,664/1,505/1,188/1,596**

**Figure 2:**
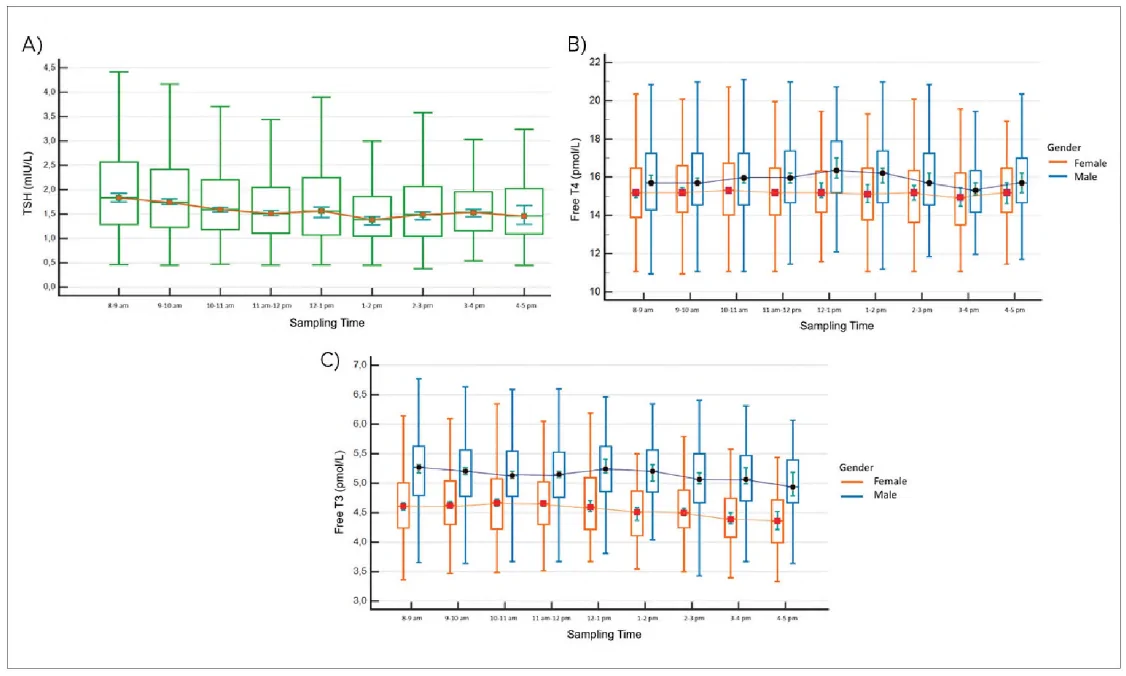
(A) TSH (mIU/L), (B) fT4 (pmol/L), and (C) fT3 (pmol/L) distributions by sampling time. Box plots displaying the minimum, maximum, lower quartile, upper quartile, and median values.

Median (2.5%-97.5%) TSH, fT4, and fT3 values for male, female, and total patients at different sampling times are presented in Table 3, Table 4, and Table 5, respectively. The distribution of parameters by sampling time is given in Figure 2.

**Table 3:** Median TSH (mlU/L) (2.5%-97.5%) values of male, female, and total patients with deviations % from the 8-9 am (Reference group) according to sampling times. Dev%: Calculated bias with reference group.<br>None of the time groups had a significant difference between genders (Z < Z*)<br>^A^ Time group that showed a statistically significant difference from the reference group (Z > Z*). ^B^ Dev%>Desirable bias (10%); <Minimum bias (15.1%)<br>^C^ Dev% > Minimum bias (15.1%)

Sampling Time	Male	Female	Total	
	Median<br>(2.5%-97.5%)	Median<br>(2.5%-97.5%)	Median<br>(2.5%-97.5%)	Dev%
8-9 am<br>(Reference group)	1.71 (0.61-3.78)	1.93 (0.71-4.18)	1.84 (0.66-4.05)	Reference
9-10 am	1.62 (0.60-3.69)	1.90 (0.69-3.99)	1.74 (0.63-3.89)	-5.43
10-11 am	1.53 (0.62-3.27)	1.68 (0.66-3.47)	1.59 (0.64-3.39)	-13.58^B^
11 am-12 pm^A^	1.41 (0.56-3.01)	1.59 (0.61-3.21)	1.51 (0.58-3.19)	-17.93^C^
12-1 pm^A^	1.49 (0.49-3.56)	1.63 (0.68-3.60)	1.56 (0.60-3.60)	-15.21^C^
1-2 pm^A^	1.34 (0.52-2.67)	1.40 (0.53-2.86)	1.39 (0.52-2.76)	-24.45^C^
2-3 pm^A^	1.39 (0.49-3.18)	1.54 (0.44-3.30)	1.49 (0.46-3.27)	-19.02^C^
3-4 pm^A^	1.50 (0.61-2.91)	1.56 (0.62-2.63)	1.53 (0.61-2.80)	-16.84^C^
4-5 pm^A^	1.32 (0.73-3.00)	1.56 (0.54-3.03)	1.46 (0.69-3.00)	-20.65^C^
**Total**	**1.53 (0.58-3.43)**	**1.72 (0.64-3.71)**	**1.61 (0.61-3.60)**	

**Table 4:** Median fT4 (pmol/L) (2.5%-97.5%) values with deviations % from the 8-9 am (Reference group) of male, female, and total patients according to sampling times. Dev%: Calculated bias with reference group<br>None of the time groups had a statistically significant difference from the reference group (Z < Z*)<br>^A^ Significant difference between genders (Z > Z*)<br>^B^ Dev% > Desirable bias (2.3%); <Minimum bias (3.5%)<br>^C^ Dev% > Minimum bias (3.5%)

Sampling Time	Male		Female		Total
	Median<br>(2.5%-97.5%)	Dev%	Median<br>(2.5%-97.5%)	Dev%	Median<br>(2.5%-97.5%)
8-9 am<br>(Reference group)	15.70 (11.97-19.82)	Reference	15.18 (11.69-19.30)	Reference	15.40 (11.86-19.79)
9-10 am	15.70 (11.71-19.82)	0	15.18 (12.08-19.30)	0	15.44 (11.97-19.56)
10-11 am	15.96 (11.97-19.90)	1.65	15.31 (11.71-19.62)	0.85	15.70 (11.97-19.82)
11 am-12 pm^A^	15.96 (12.34-19.82)	1.65	15.18 (11.72-19.05)	0	15.57 (12.09-19.44)
12-1 pm^A^	16.34 (13.01-20.33)	4.07^C^	15.18 (12.03-19.36)	0	15.70 (12.64-20.04)
1-2 pm^A^	16.21 (12.36-19.43)	3.24 ^B^	15.12 (11.85-18.78)	-0.39	15.70 (11.97-19.22)
2-3 pm	15.70 (12.58-19.95)	0	15.18 (11.83-18.93)	0	15.57 (12.09-19.56)
3-4 pm	15.31 (12.44-18.97)	-2.48^B^	14.93 (11.88-18.79)	-1.64	15.31 (12.09-18.79)
4-5 pm	15.70 (11.99-19.43)	0	15.18 (11.70-18.90)	0	15.38 (11.83-19.43)
**Total**	**15.83 (11.97-19.82)**		**15.18 (11.84-19.30)**		**15.57 (11.97-19.56)**

**Table 5:** Median fT3 (pmol/L) (2.5%-97.5%) values with deviations % from the 8-9 am (Reference group) of male, female, and total patients according to sampling times. Dev%: Calculated bias with reference group<br>None of the time groups had a statistically significant difference from the reference group (Z < Z*)<br>^A^ Significant difference between genders (Z > Z*)<br>^B^ Dev% > Desirable bias (2.4%); <Minimum bias (3.6%)<br>^C^ Dev% > Minimum bias (3.6%)

Sampling Time	Male		Female		Total
	Median<br>(2.5%-97.5%)	Dev%	Median<br>(2.5%-97.5%)	Dev%	Median<br>(2.5%-97.5%)
8-9 am^A^<br>(Reference group)	5.26 (3.97-6.42)	Reference	4.60 (3.63-5.88)	Reference	4.91 (3.73-6.30)
9-10 am^A^	5.20 (4.03-6.37)	-1.14	4.62 (3.64-5.85)	0.43	4.93 (3.77-6.29)
10-11 am^A^	5.13 (3.94-6.32)	-2.47^B^	4.66 (3.66-5.91)	1.30	4.91 (3.72-6.20)
11 am-12 pm^A^	5.14 (4.06-6.33)	-2.28	4.65 (3.70-5.80)	1.08	4.88 (3.80-6.20)
12-1 pm^A^	5.23 (3.82-6.44)	-0.57	4.59 (3.72-5.86)	-0.21	4.93 (3.78-6.19)
1-2 pm^A^	5.20 (4.10-6.26)	-1.14	4.50 (3.58-5.49)	-2.17	4.85 (3.62-6.13)
2-3 pm^A^	5.06 (3.76-6.10)	-3.80^C^	4.50 (3.67-5.48)	-2.17	4.79 (3.72-6.03)
3-4 pm^A^	5.06 (3.93-6.24)	-3.80^C^	4.39 (3.51-5.95)	-4.56^C^	4.74 (3.57-6.23)
4-5 pm^A^	4.93 (3.74-6.00)	-6.27^C^	4.36 (3.37-5.28)	-5.21^C^	4.70 (3.39-5.81)
**Total^A^**	**5.17 (3.99-6.34)**		**4.60 (3.64-5.83)**		**4.88 (3.71-6.20)**

For TSH, no significant differences were found between genders across any time groups, and evaluations were conducted on the whole group. However, fT4 results at three time points and fT3 results at all time points were significantly different (Z>Z*) between genders, so they were evaluated separately (Z and Z* values are presented in Table 6 and Table 7).

**Table 6:** Calculated Z and Z* Values in Time Groups Which Z > Z* Between Male and Female Patients for fT4 Results.

Time groups	Z	Z*
11 am-12 pm	6.23	5.79
12-1 pm	4.56	2.80
1-2 pm	4.13	3.26

**Table 7:** Calculated Z and Z* Values in Time Groups Which Z > Z* Between Male and Female Patients for fT3 Results.

Time groups	Z	Z*
8-9 am	14.60	5.83
9-10 am	17.60	7.52
10-11 am	13.86	6.57
11 am-12 pm	13.04	5.79
12-1 pm	6.90	2.80
1-2 pm	11.19	3.26
2-3 pm	11.56	4.70
3-4 pm	8.73	3.12
4-5 pm	7.07	2.27
**Total**	**35.83**	**14.94**

TSH levels decreased at subsequent sampling times compared to the 8-9 am reference group. When deviations from the reference values were compared with the recommended bias values, the deviation for the 10-11 am group was 13.58%, exceeding the desirable bias of 10%. Deviations from the reference group continued to increase over the following hours, reaching a maximum of 24.45% between 1-2 pm, which was also greater than the minimum allowable bias of 15.1%. All groups from 11 am to 5 pm also showed statistically significant differences from the 8-9 am reference group (Z and Z* values are presented in Table 8).

**Table 8:** Time Groups Which Z > Z* When Compared with the 8-9 am (reference) Group for TSH Results.

Time groups	Z	Z*
11 am-12 pm	9.28	8.22
12-1 pm	9.99	8.22
1-2 pm	9.85	6.68
2-3 pm	8.96	7.49
3-4 pm	7.49	6.61
4-5 pm	6.34	6.26

None of the fT4 and fT3 sampling time groups showed a statistically significant difference from the reference group. fT4 showed a clinically significant deviation only in males at a single time point (12-1 pm). However, fT3 values of females between 3-5 pm and males between 2-5 pm exceeded the recommended minimum bias.

## Discussion

The circadian rhythm of TSH - and, to some extent, fT4 and fT3 - is well studied in the medical literature, but very few studies have evaluated its clinical significance. In this study, a detailed retrospective analysis of hormone levels during working hours (8 am - 5 pm) was conducted, and the significance of deviations from the reference 8-9 am levels was evaluated both statistically and clinically. Our study shows a significant difference in serum TSH levels when sampling is done at different times of day.

Beginning at 8 am, TSH decreased gradually until 12 pm, reaching its lowest level between 1 and 2 pm. From 11 am onward, TSH deviations in all time groups exceeded the minimum bias values, indicating clinical significance. Additionally, statistically significant differences were observed; therefore, these results should not be compared using the same references.

In addition to testing for statistical significance, we compared deviations with the bias values of analytical performance specifications from the EFLM database to assess clinical significance. BV data serve as reference data, providing better-performing standards, and bias values based on BV are intended for laboratories to use the same reference limits.

The circadian rhythm of TSH secretion has been investigated in numerous previous studies, and a similar pattern, with a maximum between 2 and 4 am and a minimum during the daytime, has been observed [9], highlighting the importance of sampling times [10][11]. Our study demonstrated only a particular period, the working hours, of this daily rhythm with more detail and correlated well with the corresponding hours of these studies, especially for TSH. Our primary aim was not to show the circadian rhythm but to test for the significance of variations across sampling times, as in real-time practice.

Some studies have shown that thyroid function tests are affected by the seasons [9][12][13]. A study by Yamada S et al. [14] revealed that serum TSH levels exhibit seasonal variations, showing elevated levels during the winter months. Conversely, serum fT3 levels in the Japanese population were higher in the summer. Changes in fT4 levels were minimal. These seasonal fluctuations in thyroid function highlighted the importance of considering them for the accurate evaluation of thyroid function in clinical settings [14]. Therefore, in our study, we examined the homogeneity of sampling seasons across the time groups.

Food intake is reported as a source of variation in some studies, suggesting that it affects TSH measurements and leads to a gradual decrease [15][16]. This trend appears to overlap with the hormone's rhythmic pattern, but it is unclear whether food intake contributes to the TSH rhythm. Although the study group was small, Mirjanic-Azaric et al. [10] conducted a very controlled study. TSH values for the same individuals were evaluated across three experimental designs, each controlling for one of food intake, sampling hour, and intraindividual variation. Overall, the significant decrease in the two TSH levels at 90-minute intervals was due to differences in sampling time. This study also demonstrated that differences in TSH values between the two sampling times were not related to patients' fasting state [10]. Due to a lack of information on patients' fasting status at the time of sample collection, we cannot make any assumptions about it. This can be considered a limitation of our study.

In this study, unlike the statistical and clinical significance observed in TSH levels across six consecutive time intervals, fT4 levels showed only clinical significance at a single time point in males. For fT3 levels, a clinically significant difference was observed between 3-5 pm in females and between 2-5 pm in males; however, the difference was not statistically significant.

There is limited data available on T4 and T3, with controversial results. A study by Lucke reported that T4 and T3 exhibit a morning peak and an evening nadir. Since fluctuations did not exceed the normal range, they were considered insignificant in routine testing [17]. In another study, a synchronous rhythm between TSH and T3 was demonstrated, with only a relatively small change in T3 levels. This was explained by the low fraction of T3 derived from thyroid gland secretion [3]. Some investigators found no significant variation in thyroid hormones [18][19].

Thyroid hormone rhythm studies have also been reported in rats. Ikegami criticized rat experiments as not being ideal for reflecting human control mechanisms of thyroid hormone release. In this study, the author concluded that sampling time has the greatest impact on TSH levels due to its circadian rhythm [20].

RI studies [21] and BV estimations [22] focus on collecting TSH samples in the morning. The European Biodiversity Study (EuBIVAS), conducted by Bottani et al., emphasizes standardizing sampling times to 8-10 am. Circadian variations of analytes affect BV estimates, which are used to calculate RCVs [23]. If sampling times are not comparable, the clinical performances of either RIs or RCVs are questionable [4]. The Working Group on the Pre-analytical Phase in the EFLM reported that all blood sampling for routine laboratory tests should be conducted between 7 and 9 am, when circadian rhythms are at their peak [24].

Marina A. Sviridonova et al. [25], in their study of patients with hypothyroidism, found that circadian changes in TSH levels reached 73% in those with SCH. 50% of SCH cases detected in the morning could not be diagnosed in the afternoon when compared with the same RIs. They proposed a new RI for diagnosing samples taken in the afternoon (25). Thus, accurate interpretation of TSH results, in comparison with appropriate references, is essential in the clinical setting.

## Conclusion

Analysis of blood samples collected at various times of the day revealed minor fluctuations in fT4 and fT3 levels, but statistically and clinically significant variations in TSH values. For accurate clinical interpretation, the sampling time of the analyte should be comparable to that of the reference data used.

## Dodatak

### Conflict of interest statement

All the authors declare that they have no conflict of interest in this work.

### About

This study was presented as a poster at the 49^th^ Congress of the Federation of European Biochemical Societies (FEBS), 5-9 July 2025, and has been improved.
